# Connexin hemichannels and early atrophic signaling in muscle during sepsis

**DOI:** 10.3389/fphys.2025.1514769

**Published:** 2025-02-24

**Authors:** Elisa Balboa, Fujiko Saavedra, Luis A. Cea, Aníbal A. Vargas, Tomás Regueira, Juan C. Sáez

**Affiliations:** ^1^ Center for Biomedical Research, School of Medicine, Faculty of Medicine, Universidad Finis Terrae, Santiago, Chile; ^2^ Program of Reproductive Biology, Research and Innovation Center, School of Medicine, Faculty of Medicine, Universidad de los Andes, Santiago, Chile; ^3^ Instituto de Ciencias Biomédicas, Facultad de Ciencias de la Salud, Universidad Autónoma de Chile, Santiago, Chile; ^4^ Center for Integrative Biology, Faculty of Sciences, Universidad Mayor, Santiago, Chile; ^5^ Intensive Care Department, Clínica Santa María, Santiago, Chile; ^6^ Instituto de Neurociencias, Centro Interdisciplinario de Neurociencias de Valparaíso, Universidad de Valparaíso, Valparaíso, Chile

**Keywords:** resting membrane potential, muscles, inflammation, channeloapthy, connexin

## Abstract

Sepsis pathogenesis is complex, and effective treatments are limited, leading to high mortality rates between 20% and 55%. Early identification of factors contributing to sepsis-related muscle dysfunction is critical for risk stratification and potential therapeutic development. The immune response during sepsis affects skeletal muscles, contributing to organ dysfunction and worsening prognosis. In this study, we explore the role of connexin hemichannels (Cx HCs) in the early changes in muscle homeostasis during sepsis. Using a cecal ligature and puncture (CLP)-induced sepsis model, we assessed IL-6 levels, weight loss, myofiber cross-sectional area, resting membrane potential, and connexin expression in control and Cx43/Cx45-deficient mice. CLP induced IL-6 elevation, sarcolemma permeabilization, reduced membrane potential, and activation of the ubiquitin–proteasome pathway in control mice, while Cx43/45-deficient mice exhibited reduced all CLP-induced muscle alterations. These findings suggest that Cx43 and Cx45 are involved in the early development of muscle alterations during sepsis.

## Graphical Abstract

**Figure:**
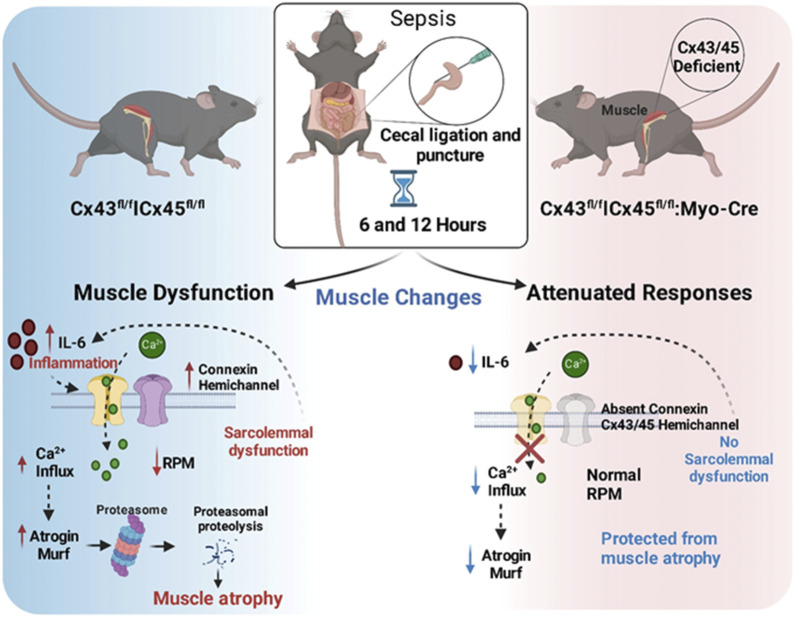
Schematic Representation of Early Events in the CLP-Induced Sepsis Model and the Role of Connexins in Muscle Atrophy.

## Introduction

Severe sepsis is the leading cause of death in Intensive Care Units (ICUs) world-wide, with mortality rates ranging from 20% to 55% (Bauer et al., 2020; Prest et al., 2022). In Chile, severe sepsis is highly prevalent in ICUs, with a case-fatality rate of 26.7%, highlighting its significant impact both nationally and globally (Dougnac et al., 2007).

Muscle atrophy is a significant consequence of sepsis, is highly prevalent and associated with high mortality (Fleischmann-Struzek et al., 2020; Singer et al., 2016). The main cause of death during sepsis is multiorgan failure (MOF), with muscle dysfunction being one of the key components (Bilevicius et al., 2001; Khan et al., 2008). Both respiratory and peripheral skeletal muscles are adversely affected by the systemic inflammatory response during acute and late sepsis (De et al., 2002; Callahan and Supinski, 2009). This muscle dysfunction is closely linked to a prolonged stay in the ICU and the need for mechanical ventilation, leading to higher mortality rates and long-term mobility issues (Hermans and Van den Berghe, 2015). Sepsis-associated muscle atrophy is characterized by a reduction in force-generating capacity and muscle wasting due to increased proteolysis over protein synthesis (Eikermann et al., 2006; Tiao et al., 1994; Lang et al., 2007). Several pathogenic mechanisms have been proposed to explain these changes, including mitochondrial dysfunction (MD) (Brealey et al., 2004), microvascular alterations, metabolic derangements, and oxidative stress (Prauchner, 2017; Wasyluk and Zwolak, 2021). Additionally, the sarcolemma becomes electrically unexcitable (Trojaborg et al., 2001; Fernández-Lorente et al., 2010) in the presence of elevated sarcoplasmic Ca^2+^ concentrations (Baracos et al., 1986), suggesting that acquired channelopathy may play a role in the muscle dysfunction observed during sepsis.

In our previous studies on late sepsis induced in mice, we showed evident skeletal muscle atrophy induced by cecal ligation and puncture (CLP), which was associated with the *de novo* expression connexin 39, 43, and 45 hemichannels (Cx HCs) and P2X7 receptors (P2X7Rs) in the skeletal myofibers. These findings were associated with an increased sarcolemma permeability, an elevated intracellular free Ca^2+^ concentration, and the activation of protein degradation via the ubiquitin–proteasome pathway (Balboa et al., 2018). In addition, we described a moderate reduction in the mitochondrial oxygen consumption, a reduced mitochondrial membrane potential, and increased mitochondrial superoxide production (Balboa et al., 2018). Studies on the skeletal muscles of animals undergoing endotoxemia have also unveiled atrophy associated with the *de novo* expression of poorly selective membrane channels, including Cx HCs and P2X7Rs (Cea et al., 2019). Moreover, we found that the myofibers of mice deficient in Cx43 and Cx45 expression showed drastic resistance to a lipopolysaccharide- or denervation-induced reduction in the resting membrane potential, sarcolemma permeabilization, and increases in the sarcoplasmic Ca^2+^ concentrations in muscle fibers (Cea et al., 2019). Similar muscle protection was found in denervation or glucocorticoid-induced muscle atrophy (Cea et al., 2013; Cea et al., 2016), suggesting that the expression of non-selective channels is common in muscle atrophy of different etiologies.

Given that Cx HC expression worsens the pathological conditions of various skeletal muscles and occurs within the first hours after the onset of these pathologies (Cea et al., 2019; Cea et al., 2016), we decided to test how early Cx HC expression occurs following sepsis and whether it happens before the muscle atrophy associated with muscle protein degradation. We hypothesized that Cx43/Cx45 skeletal-myofiber-deficient animals may be protected from developing muscle depolarization and from the activation of protein degradation pathways induced by sepsis. Using a well-characterized model of sepsis (Balboa et al., 2018), we found that skeletal muscles express *de novo* Cx39, 43, and 45 as well as P2X7Rs as early as 6 h after the beginning of sepsis, and that these are related to a reduction in the resting membrane potential and the activation of protein degradation. These findings could be relevant for future interventions and for developing novel therapeutic strategies for preventing sepsis-induced skeletal muscle atrophy.

## Methods

### Reagents

HEPES, leupeptin, pepstatin, phenylmethylsulphonyl fluoride (PMSF), N-benzyl-ptoluene (BTS), sulfonamide, collagenase type I, suramin, and ethidium (Etd^+^) bromide were obtained from Sigma-Aldrich (St. Louis,MO, United States). The DMEM/F12 culture medium and fetal bovine serum albumin were from Gibco (Mary-land, United States). Tramadol was obtained from Genfar (Gentilly, Francia). Previously characterized anti-Cx39 and anti-Panx1 antibodies were used (Riquelme et al., 2013). Anti-atrogin1, anti-Murf, anti-Cx45, anti-Cx43, and P2X7R antibodies were obtained from Abcam (Cambridge, UK). Cy2-and Cy3-conjugated goat anti-rabbit IgG antibodies were purchased from Jackson Immuno Research (Indianapolis, IN, United States).

### Animals

The present study was performed with approval from the bioethics committee of Pontificia Universidad Católica de Chile (No. 150323001). We used C57BL6 skeletal-muscle-deficient mice for Cx43 and Cx45 generated from breeding Cx43^fl/fl^ mice and Cx45^fl/fl^ mice with Myo-Cre mice, which express Cre recombinase under the control of a myogenin promoter and the MEF2C enhancer (Cea et al., 2013; Cisterna et al., 2016). The Cx43^fl/fl^Cx45^fl/fl^ mice (hereafter referred to as Cre-) normally express Cx43 and Cx45, and the Cx43^fl/fl^Cx45^fl/fl^:Myo-Cre mice are double-inducible deficient in Cx43 and Cx45 (hereafter referred to as Cx3/45 deficient mice). The mice were kept in standard housing conditions with a 12-h/12-h dark/light cycle and food and water *ad libitum*. To minimize variability, only male mice were used in this study, focusing on sex-based differences in skeletal muscle physiology (Haizlip et al., 2015).

The tibialis anterior (TA) muscle and diaphragm were used for hematoxylin-eosin staining and immunofluorescence. For isolated fiber measurements, the flexor digitorum brevis (FDB) muscle was chosen due to the ease of isolating intact fibers without damage. Using different mouse muscles for distinct methodologies helps minimize the number of animals required. Importantly, both the FDB and TA muscles are composed predominantly of fast-twitch fibers, sharing similar metabolic and contractile properties, which supports the comparability of findings across these muscles.

### Sepsis induction by cecal ligation and puncture

The mice were placed under CLP for six or 12 h. For this, the mice were anesthetized using isoflurane (Baxter Healthcare, Guayama, Puerto Rico). The abdomen was shaved, and a midline incision (0.5 cm) was made in the intraperitoneal area. The cecum was isolated, tied, and punctured once with a 21-gauge needle (0.8 × 38 mm), and a small amount of cecal material was extruded. The cecum was returned to the abdomen, and 200 μL of PBS 1X was added to ensure sepsis. The incision was closed with a 20 mm nylon surgical suture (Tagumedica S.A., Santiago, Chile). Sham controls were subjected to the same surgical protocol and cecal isolation, but the cecum was neither tied nor punctured. The control mice consisted of the sham group. A sham procedure was performed for each time point (0, 6, and 12 h). However, no significant differences were observed between the sham groups at different time points for the parameters analyzed. Therefore, to minimize the number of mice used and to increase the sample size of the control group, all the sham groups were combined to form a single control group.

### Mouse IL-6 immunoassay

The mouse IL-6 content was measured in 35 μL of plasma using the Mouse IL-6 Quantikine ELISA Kit (R&D Systems, Inc; Minneapolis, MN, United States), according to the manufacturer’s instructions.

### Histological analysis

Freshly dissected TA muscles were embedded in a tissue-mounting OCT solution (Andes import, Chile) and fast frozen in liquid-nitrogen-cooled isopentane (Merck, Germany). Serial cryostat sections with a 16 μm thickness were obtained, placed on glass slides (75 × 25 mm, B&C, Germany), and fixed for 10 min with 4% (wt/vol) paraformaldehyde (Electron Microscopy Sciences, United States) for immunofluorescence or cross-sectional area (CSA) analyses.

### Cross-sectional area

The muscle fiber size was quantified by evaluating the cross-sectional area (CSA) of the fibers, measured as described previously (Cea et al., 2013). Briefly, cross-sections were stained with hematoxylin and eosin (H&E). The CSA was evaluated in multiple fibers from three images per mouse (4 mice per group) by using offline analyses with the ImageJ software (National Institutes of Health).

### Immunofluorescence

To detect different proteins in cross-sections (16 μm), the TA muscles sections were blocked for 1 h in a blocking solution (1% BSA, 50 mM NH4Cl, 0.01% Triton x-100, 1X PBS, and a pH of 7.4) and then incubated at 4°C overnight with diluted primary anti-atrogin1 (1:300), anti-Murf 1 (1:300), anti-Cx39 (1:300), anti-Cx43 (1:300), anti-Cx45 (1:300), anti-Panx1 (1:300), or anti-P2X7 (1:200) antibodies (Balboa et al., 2018). The samples were washed 4 times with PBS 1X and then incubated with an appropriate dilution of Cy2-or Cy3-conjugated goat anti-rabbit IgG antibodies (1:300) for 1 h in a dark room. The samples were washed 4 times with PBS 1X and one time with distilled water, and then mounted using Fluoromount-G™ with DAPI (Electron Microscopy Science, Hatfield, PA, United States) on glass slides. The images were generated using a Nikon Eclipse Ti micro-scope, and the fluorescence intensity was quantified with ImageJ. For each condition, histological sections from at least four mice were analyzed, with a minimum of three histological sections per mouse. For each section, three photographs were taken, and the fluorescence intensity of each photograph was quantified. The average fluorescence intensity for each image was plotted using a violin plot.

### Isolation of skeletal myofibers

The myofibers of the mouse FDB muscles were isolated from anesthetized and sacrificed mice, as described previously (Riquelme et al., 2013). The muscles were immersed in a cultured medium (DMEM/F12 supplemented with 10% fetal bovine se-rum) containing 0.2% collagenase type I, and incubated for 2.5 h at 37°C. Then, they were transferred to a 15 mL tube containing 3 mL of Krebs buffer (in mM: 145 NaCl, 5 KCl, 3 CaCl_2_, 1 MgCl_2_, 5.6 glucose, 10 HEPES-Na, and a pH of 7,4) plus 10 μM BTS (contraction inhibitor, to reduce muscle damage). The muscle tissue was incubated for 2 min with 200 μM suramin, a P2 receptor inhibitor, to prevent the effects of ATP release during the procedure. Then, the tissue was gently triturated by passing it 7 times through a wide-tip Pasteur pipette. The myofibers were left to decant for 7 min, and the sediment was washed with HEPES-buffered Krebs saline solution containing 10 μM BTS. The myofibers were gently triturated again by passing them 15 times through a narrow tip to dissociate single myofibers. The dissociated myofibers were left to decant again for 7 min, after which the sediment was washed, resuspended in HEPES-buffered Krebs saline solution containing 10 μM BTS, and placed in 1.5 mL Eppendorf tubes until use for further analyses.

### Dye uptake assay

The myofiber permeability was evaluated by assessing the ethidium (Etd^+^) uptake using time-lapse measurements, as described previously (Riquelme et al., 2013). In brief, freshly FDB myofibers plated onto plastic culture dishes were washed twice with Krebs buffered solution. For the time-lapse measurements, the myofibers were incubated in a recording Krebs solution containing 5 μM Etd^+^ and recorded for 5 min in a control solution. They were subsequently recorded for 5 min after an HC blocker (lanthanum ion (La^3+^), 200 µM) was added to the Krebs solution (22). The Etd^+^ fluorescence was recorded in regions of interest (ROI) corresponding to the nuclei of the myofibers, using a Nikon Eclipse Ti inverted microscope and the NIS-Element software (Nikon, Tokio, Japan).

### Oxygen consumption

The oxygen consumption of muscle fibers was measured as described previously (Kuznetsov et al., 2008). Fiber bundles from the soleus muscle (0.2–0.8 mg dry/wet) were separated along their longitudinal axis with a pair of needle-tipped forceps under magnification with an Olympus SZ61 microscope. The bundles were then treated with 50 μg/mL of saponin for 30 min in ice and subsequently washed in an MIR05 medium. High-resolution O^2^ consumption measurements were conducted at 30°C with the OROBOROS Oxygraph-2K (OROBOROS Instruments, Innsbruck, Austria).

### Resting membrane potential (RMP)

The mice were anesthetized with 70 µL of a mixture consisting of xylazine and ketamine (1:3 proportion), and a conventional *in vivo* microelectrode recording technique was used (Cisterna et al., 2020). The flexor digirotium brevis (FDB) muscle was completely exposed and submerged in Krebs solution (pH of 7.4), and the RMP measurement was set to “current clamp” at 37°C. The Ag-Cl reference electrode was inserted in a 3 M KCl agarose bridge and submerged in Krebs solution to record superficial fibers from the FDB muscle. Each electrode was fabricated from a borosilicate capillary (A-M System, Inc., Cat # 596800) with the horizontal stretcher P97 (Sutter instruments, CA, EUA), and filled with a 3 M KCl solution with electrode resistances within the range of 40–50 MΩ. The recorded RMP corresponded to the potential value measured when the microelectrode accessed the sarcoplasm, crossed the sarcolemma, and remained stable for 5 s after impelling. All the experiments used an Olympus IX 51 inverted microscope with an Axopatch1-D amplifier (Molecular Devices, CA, EUA), a Digidata 1,322 digitalizer, and the Clampex 9.1 acquisition software.

### Statistics

Based on the literature and our previous studies, we calculated the required sample size to detect significant differences in various parameters with a significance level of 0.05 and a power of 0.8, ensuring the minimum number of animals needed to observe significant effects, which was determined to be five in most cases.

The data for each condition are summarized in different type graphs as means ± standard error (SEM), where the number of mice per treatment group is indicated in the legend of each figure. The data were evaluated using non-parametric tests, specifically the Kruskal–Wallis test followed by Dunn’s multiple comparison test. Analyses were carried out using the GraphPad software. Significant differences between groups were considered when P < 0.05.

## Results

### Characterization of the CLP-Induced sepsis model in early stages

**FIGURE 1:**
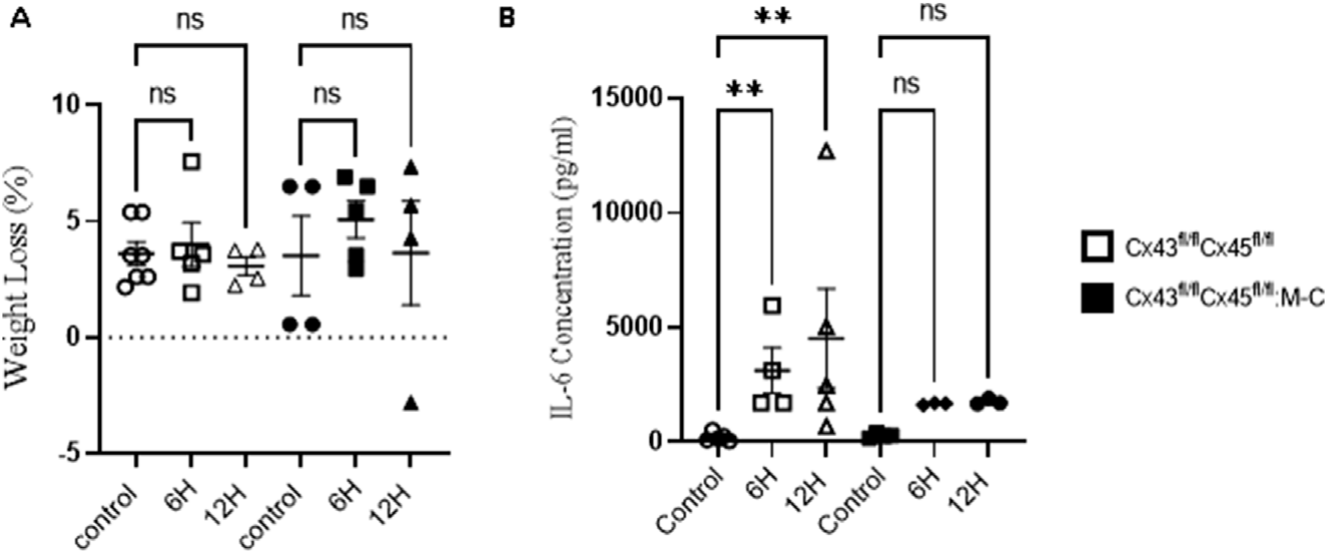
**(A)** Animal Weight Changes after CLP-Induced Sepsis. Body weight over time in control and at 6 and 12 h after CLP, a sepsis model. Body weight loss in Cx43fl/flCx45fl/fl and Cx43fl/flCx45fl/fl:M-C (Cx43/45 deficient) mice. **(B)** Quantification of plasma IL-6 concentration in control and CLP mice at 6 and 12 h after CLP. The cytokine was quantified using the Mouse IL-6 Quantikine ELISA Kit (R&D Systems, Inc; Minneapolis, United States). Statistical comparisons were made with the GraphPad Prism software using non-parametric tests, specifically the Kruskal–Wallis test followed by Dunn’s multiple comparison test. The error bars represent the standard error of the mean (SEM). *p < 0.05, **p < 0.01, ns: not significant. n = at least four for each condition.

Abundant evidence indicates that sepsis induces a myopathy characterized by reductions in muscle mass (Rocheteau et al., 2015). However, the mechanisms of this are not well understood. Previously, we observed that WT mice showed an almost 17% reduction in body weight 2 days after CLP, and this percentage remained until day 5, finally recovering at day 7 (Balboa et al., 2018). This weight reduction was attributed to the loss of muscular mass due to the documented muscular atrophy. However, at early time points, a decrease in mouse weight could have been due to a postoperative effect stemming from reduced water or food intake as a result of postoperative discomfort. The observed weight changes were similar between the control (sham) animals and those subjected to CLP in Cre- (Cx43^fl/fl^Cx45^fl/fl^) and Cx43/45-deficient (Cx43^fl/fl^Cx45^fl/fl^: Myo-Cre) mice at 6 and 12 h (Figure 1A). At 48 h, the mice begin to experience significant weight loss, which is attenuated in Cx43/45-deficient mice (Supplementary Figure 1).

As previously demonstrated, skeletal muscle IL-6 production contributes to the overall circulating IL-6 levels during exposure to a septic peritoneal infection (Laitano et al., 2021). We aimed to evaluate whether differences in the IL-6 levels existed in our model. As shown in Figure 1B, a tenfold increase in the IL-6 levels was observed in Cre-mice at 6 h post-CLP compared to the controls, and this increased approximately tenfold at 12 h. Conversely, in Cx43/45-deficient mice, only a fivefold increase in the IL-6 levels was observed at 6 and 12 h post-CLP, suggesting that there could be less muscle inflammation in Cx43/45-deficient mice. Hematoxylin and eosin staining revealed immune cell infiltration in muscles of Cre-mice at 12 h post-CLP, with multiple infiltration foci observed. In contrast, muscles of Cre + mice exhibited very few foci of immune cell infiltration, further supporting the observation of reduced muscle inflammation in these mice (Supplementary Figure 2C).

### Upregulation of ubiquitin–proteasome degradation proteins in peripheral and diaphragm muscles during CLP-induced sepsis at 6–12 h

**FIGURE 2:**
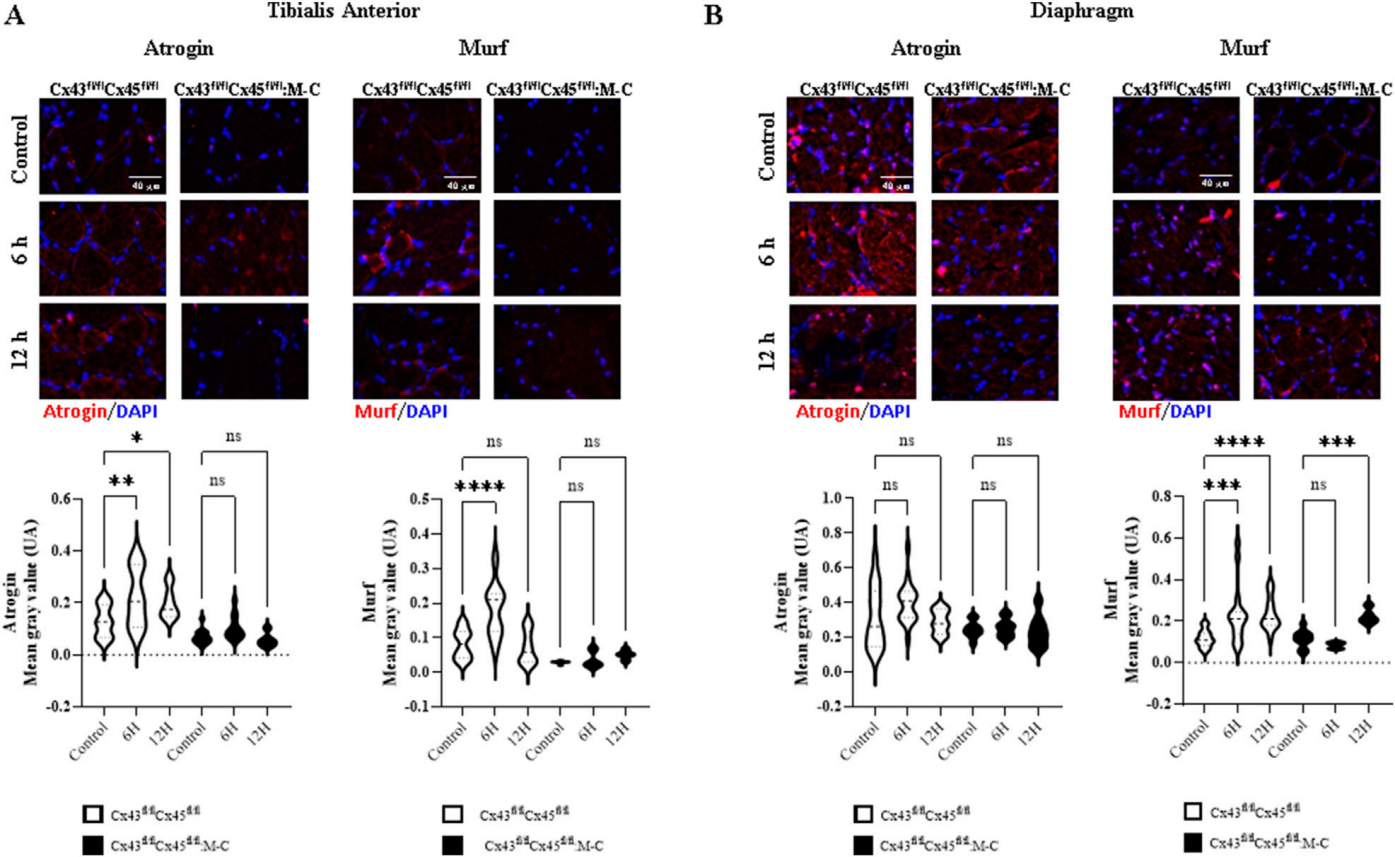
CLP-induced sepsis increases the immunoreactivity of atrogin-1 and Murf. Immunofluorescence over time was evaluated in control mice and at 6 and 12 h after CLP. **(A)** Representative images and quantification of immunofluorescence for atrogin-1 and Murf in tibialis anterior muscle sections from control and CLP mice at different time points post-CLP. **(B)** Representative images and quantification of immunofluorescence for atrogin-1 and Murf in diaphragm muscle sections from control and CLP mice at different time points post-CLP. For each condition, histological sections from at least four mice were analyzed, with a minimum of three histological sections per mouse. Fluorescence intensity was quantified from each section, and the average fluorescence intensity for each image was plotted using a violin plot. Statistical comparisons were performed using GraphPad Prism software, with non-parametric tests (Kruskal–Wallis test followed by Dunn’s multiple comparison test). Error bars represent the standard error of the mean (SEM) (*p < 0.05, **p < 0.01, ***p < 0.001).

As we have previously shown that sepsis induces a decrease in CSA at 7 days (Balboa et al., 2018), we wanted to evaluate the CSA at early times. At 6 and 12 h post-sepsis induction, we observed no significant changes CSA (Supplementary Figure 2); however, levels of atrogins and MuRF, proteins associated with muscle atrophy (Tiao et al., 1994), were increased in Cre-mice. Notably, this increase was not observed in deficient CXs43/45 mice (Figure 2A). On the other hand, the diaphragm muscles showed an increase in the Murf immunoreactivity at 6 and 12 h after CLP in Cre-mice and at 12 h in Cx43/45 deficient mice (Figure 2B).

### Early increase in Cx39, Cx43, and Cx45 hemichannel expression and decrease in resting membrane potential in peripheral and diaphragm muscles following CLP-induced sepsis

Previous studies have described that denervated skeletal muscles show the *de novo* expression of Cx39, 43, and 45 HCs, in addition to P2X7Rs, and the upregulation of pannexin1 (Panx1), which play a relevant role in muscle atrophy (Cea et al., 2013; Cisterna et al., 2016). Nevertheless, there is currently no evidence regarding the early expression of membrane channel-forming proteins in the skeletal muscles of mice subjected to CLP. To study this possibility, we evaluated the reactivity of Cx39, 43, and 45, Panx1, and P2X7Rs in slices of the TA and diaphragm muscles from Cre- and Cx43/45-deficient mice subjected or not to CLP.

**FIGURE 3:**
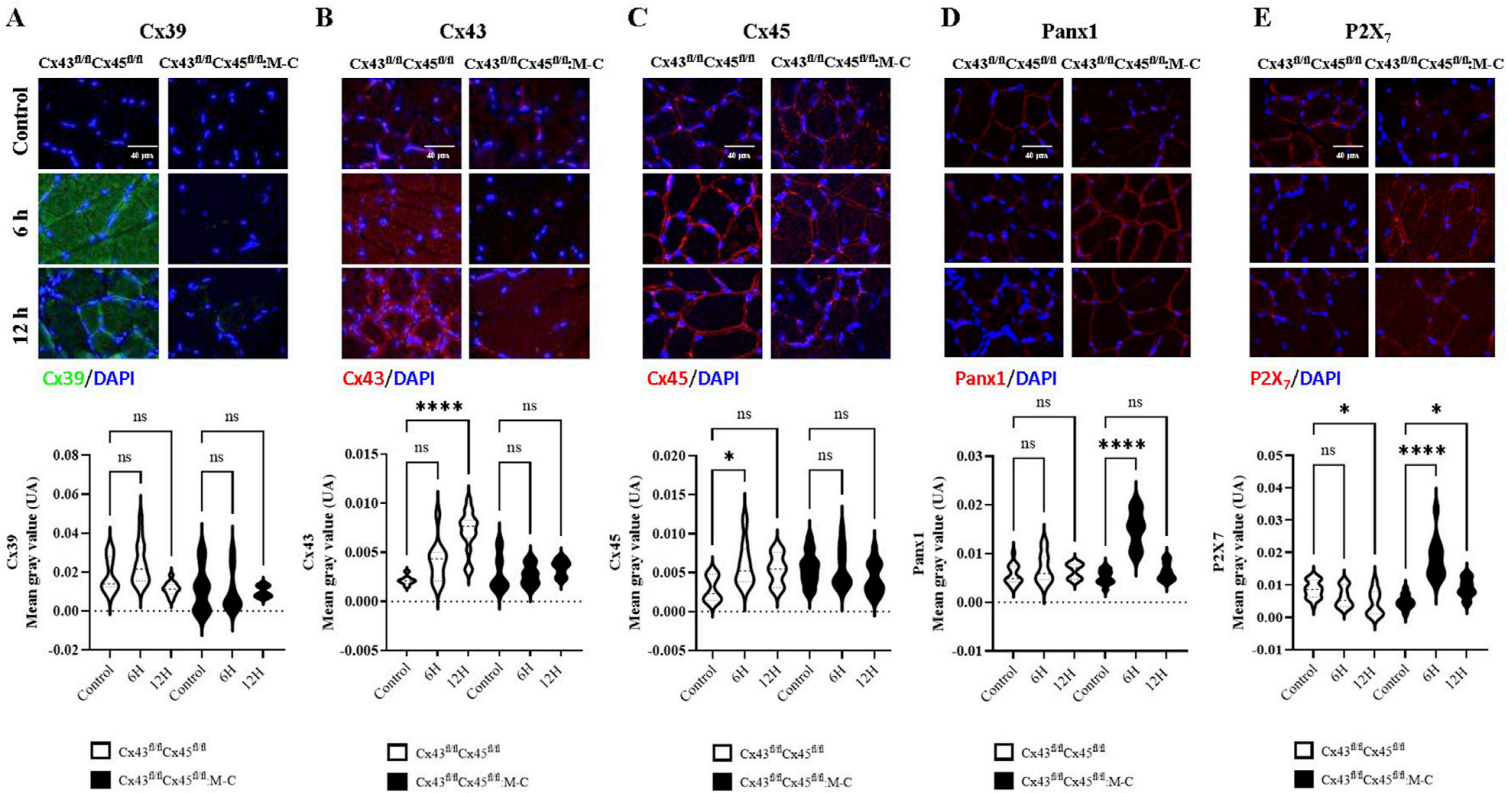
Cx43/45-deficient mice are protected against the sepsis-induced *de novo* expression of connexin hemichannels in the sarcolemma of skeletal muscle. Representative images and quantification of immunofluorescence for **(A)** Cx39, **(B)** Cx43, **(C)** Cx45, **(D)** Panx1, and **(E)** P2X7Rs in tibialis anterior muscle sections from control and CLP mice at different time points post-CLP, for Cre- (Cx43^fl/fl^Cx45^fl/fl^) and Cx43/Cx45-expression-deficient (Cx43^fl/fl^ Cx45^fl/fl^) mice. Fluorescence intensity was quantified in at least three histological sections analyzed from a minimum of four mice per treatment group. The data is presented using box and violin plots. Statistical comparisons were performed using GraphPad Prism software with non-parametric tests (Kruskal–Wallis test followed by Dunn’s multiple comparison test). Error bars represent the standard error of the mean (SEM) (*p < 0.05, **p < 0.01, ***p < 0.001).

In the TA muscles of Cre-mice Cx45 increased at 6 h, and Cx43 increased at 12 h. Meanwhile, Panx1 showed no significant changes, and P2X7Rs decreased at 12 h. In contrast, no changes were observed in Cx43/45-deficient mice in the expression of Cx39, Cx43, and Cx45 at both 6 and 12 h post-CLP. However, Panx1 and P2X7Rs increased at 6 h post-CLP (Figures 3A–E). We found similar changes in the diaphragm muscles to those observed in the TA muscles (Supplementary Figure 3). All the proteins analyzed were mainly detected in the contours of the myofibers, and were likely associated with the sarcolemma.

**FIGURE 4:**
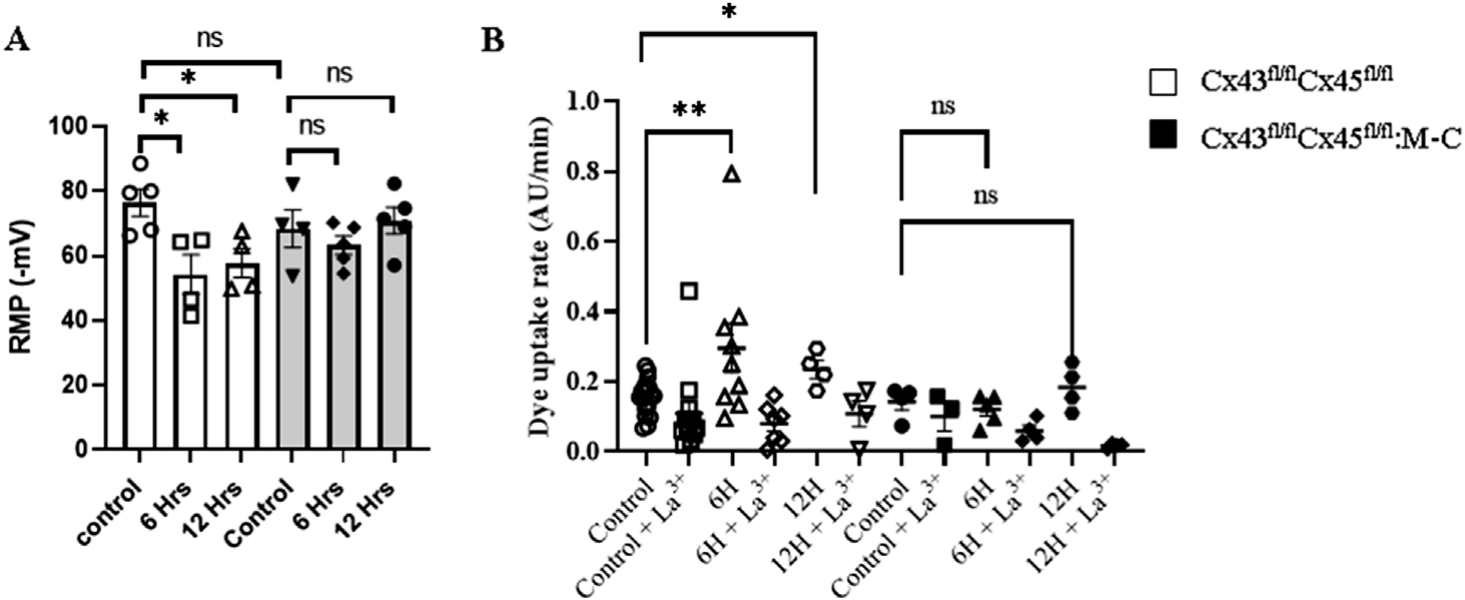
Sepsis Augments Connexin Hemichannel Aperture Probability, which is Prevented in Cx43/45 deficient mice. **(A)** Resting membrane potential (RMP) was evaluated at 0, 6 and 12 h of CLP in control and Cx43/45 deficient mice. **(B)** The permeability of the sarcolemma was measured in time-lapse experiments of ethidium (Etd^+^) uptake in isolated skeletal myofibers from the FDB muscle of control and CLP mice after 6 and 12 h. Ethidium uptake was assessed in at least five isolated fibers from a minimum of four mice for each treatment group. Statistical comparisons were conducted by utilizing GraphPad Prism software and non-parametric tests, specifically the Kruskal–Wallis test followed by Dunn’s multiple comparison test. The error bars represent the standard error of the mean (SEM) (*p < 0.05, **p < 0.01, ***p < 0.001).

Given that the *de novo* expression of connexins after CLP was observed in the myofibers of Cre-animals, we aimed to assess whether the expression of these connexins on the sarcolemma had an impact on the resting membrane potential (RMP) of myofibers due to an increase in the ion flux through the HCs, as described before (Cisterna et al., 2020). Furthermore, the sarcolemma of the skeletal myofibers in mice exposed to sepsis becomes electrically unexcitable (Trojaborg et al., 2001). Accordingly, we evaluated whether or not the RMP is altered during early sepsis in Cre- and Cx43/45-deficient mice subjected to CLP. We observed that the RMP in Cre-animals decreased significantly at 6 h and 12 h after CLP. In contrast, Cx43/45 animals were protected, and their RMP was kept stable over time (Figure 4A).

Cx HCs allow for the flow of ions such as Na^+^ and Ca^2+^ [24], and given our previous results indicating that Cx HCs were expressed in the muscles and the RMP was altered in CLP Cre-mice, we decided to evaluate the Cx HC activity in freshly isolated FDB myofibers using the ethidium (Etd+) uptake technique. We found that the Etd^+^ uptake increased in septic Cre-animals as early as 6 h compared with control animals, but it did not increase in septic Cx43/45-deficient animals. In addition, the Etd^+^ uptake was reverted by adding La^3+^, a non-selective Cx HC blocker (Shoji et al., 2014). These results suggest that sepsis increases the sarcolemma permeability through Cx HCs (Figure 4B).

### CLP induces similar changes in the mitochondrial oxygen consumption rate and the ex-pression of mitochondrial respiratory chain components in both Cre- and Cx43/45-deficient mice

Changes induced by acute inflammatory conditions, such as sepsis, are linked to the dysfunction of mitochondrial components in muscle [30]. Moreover, alterations in the sarcolemma permeability and RMP secondary to ion influx, such as Ca^2+^, are important for the correct functioning of mitochondria (Ruiz-Meana et al., 2009; Contreras et al., 2010). To determine the mitochondrial function in CLP mice, we measured the mitochondrial oxygen consumption rate (OCR) using permeabilized fibers from the soleus muscles. The mitochondrial oxygen consumption rate was similar between Cre- and Cx43/45-deficient mice across all the evaluated parameters (Supplementary Figure 4). CLP at 6 and 12 h did not affect the mitochondrial function in our model. This suggests that mitochondria can withstand the changes observed in the other results.

## Discussion

In the present study, which was carried out on an early model of sepsis, connexin expression was observed to occur early during sepsis and was functionally active as HCs and associated with sarcolemma depolarization, as well as the activation of muscle protein degradation pathways. We did not observe mitochondrial dysfunction (MD), suggesting that MD can occur later or is a downstream phenomenon of sarcolemma Cx HC expression.

IL-6 increased with CLP, being more pronounced in Cre-mice than in Cx43/45-deficient mice. Although this inflammation was lower in myofiber Cx43/45-deficient mice, it was not absent. It should be noted that this measurement was performed in plasma, thus representing systemic inflammation induced by sepsis. Activated macrophages, as well as other cell types, can generate IL-6 and contribute a significant amount of pro-inflammatory cytokines, including IL-6. However, given that Cx43/45-deficient mice were muscle-specific, the decrease in inflammation suggests that there is less inflammation at the muscular level. It should be highlighted that the non-expression of connexins at the muscular level impacted systemic inflammation. This suggests that skeletal myofibers participate by orchestrating a relevant phenomenon in systemic inflammation induced by CLP or that the muscle itself becomes inflamed, and the non-expression of connexin protects the muscle from this inflammation. The expression of connexins by myofibers could have resulted from the effect of pro-inflammatory mediators, as previously shown in freshly isolated myofibers treated with TNF-α plus IL-1β (Cea et al., 2019).

Changes in the total body weight (BW) can influence the gain and loss of muscle mass (McNair et al., 2020). We observed that, in CLP mice, weight loss began at 48 h (Supplementary Figure 1), which could not be explained by a decreased food intake. Again, considering the myofiber specificity of Cx43/45 muscle-deficient mice, the non-expression of connexins could protect them against weight loss, given their effect on the muscle. However, we hypothesize that decreases in muscle mass begin later than 24 h, as this involves processes that require more time to occur.

Since 6 and 12 h is a very early time point to observe a decrease in CSA, we did not expect to see differences between Cre- and Cx43/45-deficient mice. However, we analyzed the activation of the protein degradation cascade induced by the Atrogin and Murf proteins and found that they were elevated at 6 h post-CLP in Cre-mice. Notably, these proteins were not elevated in the TA muscle from Cx43/45-deficient mice, suggesting that the expression of connexins in the sarcolemma induced the activation of this pathway. This may be explained by reductions in the myofiber RMP observed in these mice, which are associated with ion influx and could contribute to the activation of the ubiquitin–proteasome pathway. In addition, the influx of Ca^2+^ could be responsible for the induction of Murf and Atrogin expression and activation (Ham et al., 2014).

On the other hand, what we did not expect was the elevated Murf1 levels observed in the diaphragm. At 12 h, Murf1 expression was significantly increased in the diaphragm of Cx43/45-deficient mice, which we consider an unexpected result. This finding could potentially be related to the increased expression of Panx1, the different fiber composition in the diaphragm compared to the TA, or other unidentified factors. Further studies are required to determine the underlying mechanisms responsible for this differential response between the diaphragm and the TA muscle in Cx43/45-deficient mice.

Considering that all the connexin proteins observed using immunofluorescence were in the periphery of the cells, their presence on the sarcolemma can be suggested. Furthermore, given that cells from septic Cx43/45-deficient mice did not show permeability to Etd^+^ and cells from Cre-mice were permeable to Etd^+^, which was completely blocked by La^3+^, the increase in the sarcolemma permeability was likely primarily mediated by Cx HCs. Neither Panx1 nor P2X7Rs were blocked by La^3+^ (Shoji et al., 2014), suggesting that only Cx HCs, which are absent in normal muscles as reported previously (Cea et al., 2013; Riquelme et al., 2013; Cisterna et al., 2016), are the main contributors to the increase in the sarcolemma permeability. This increase in the sarcolemma permeability could also largely explain the RMP reduction and reduced excitability of myofibers. The observed upregulation of P2X7R and persistent expression of Panx1 in the muscle tissues of Cx43/45-deficient mice observed at 6 h CLP likely reflects a multifaceted response. Given the absence of Cx43-and Cx45-based hemichannels, the increase in P2X7R and Panx1 expression may represent a systemic response to sepsis, as both proteins are known to be upregulated during inflammatory conditions (Cea et al., 2019) and contribute to immune cell recruitment and inflammasome activation (Loureiro et al., 2022; Chen et al., 2023). Further studies are required to understand this response better.

The peripheral muscles of septic patients have been described as showing signs of bioenergetic failure, comprising oxidative stress, MD, and ATP depletion (Baracos et al., 1986). In our model, we did not observe MD in the early stages of sepsis (6 and 12 h post-CLP), strongly suggesting that sarcolemma channelopathy occurs before MD. It is possible that the loss of the electrochemical gradient across the sarcolemma, caused by the expression of functional, poorly selective sarcolemma channels, could reduce the mitochondrial membrane potentials (MMPs). The latter is greatly affected by increases in the intracellular Na^+^ or Ca^2+^ signals, but MD manifests later, based on our observations, only becoming apparent at 48 h (Supplementary Figure 2). Our findings highlight the potential role of sarcolemma channelopathy in the early pathogenesis of sepsis-induced muscle dysfunction. Furthermore, the protective effect observed in Cx43/45-deficient mice, both in terms of inflammation and muscle preservation, suggests that targeting sarcolemma Cx HCs could be a potential therapeutic strategy for mitigating sepsis-induced muscle pathology. The sued of selective inhibitors of Cx HCs such as D4 molecule (Cisterna et al., 2020), Gap-19 or Gap-26 could be considered for therapeutical use.

## Data Availability

The raw data supporting the conclusions of this article will be made available by the authors, without undue reservation. It can be accessed on Figshare repository, https://figshare.com/s/7fc1c880ef780798bb23.

## References

[B1] Balboa E. Saavedra-Leiva F. Cea L. A. Vargas A. A. Ramirez V. Escamilla R. (2018). Sepsis-induced channelopathy in skeletal muscles is associated with expression of non-selective channels. Shock 49 (2), 221–228. 10.1097/SHK.0000000000000916 28562477

[B2] Baracos V. Greenberg R. E. Goldberg A. L. (1986). Influence of calcium and other divalent cations on protein turnover in rat skeletal muscle. Am. J. Physiol. 250 (6 Pt 1), E702–E710. 10.1152/ajpendo.1986.250.6.E702 3521317

[B3] Bauer M. Gerlach H. Vogelmann T. Preissing F. Stiefel J. Adam D. (2020). Mortality in sepsis and septic shock in Europe, North America and Australia between 2009 and 2019— results from a systematic review and meta-analysis. Crit. Care 24 (1), 239. 10.1186/s13054-020-02950-2 32430052 PMC7236499

[B4] Bilevicius E. Dragosavac D. Dragosavac S. Araújo S. Falcão A. L. Terzi R. G. (2001). Multiple organ failure in septic patients. Braz J. Infect. Dis. 5 (3), 103–110. 10.1590/s1413-86702001000300001 11506772

[B5] Brealey D. Karyampudi S. Jacques T. S. Novelli M. Stidwill R. Taylor V. (2004). Mitochondrial dysfunction in a long-term rodent model of sepsis and organ failure. Am. J. Physiol. Regul. Integr. Comp. Physiol. 286 (3), R491–R497. 10.1152/ajpregu.00432.2003 14604843

[B6] Callahan L. A. Supinski G. S. (2009). Sepsis-induced myopathy. Crit. Care Med. 37 (10 Suppl. l), S354–S367. 10.1097/CCM.0b013e3181b6e439 20046121 PMC3967515

[B7] Cea L. A. Balboa E. Puebla C. Vargas A. A. Cisterna B. A. Escamilla R. (2016). Dexamethasone-induced muscular atrophy is mediated by functional expression of connexin-based hemichannels. Biochim. Biophys. Acta 1862 (10), 1891–1899. 10.1016/j.bbadis.2016.07.003 27437607

[B8] Cea L. A. Balboa E. Vargas A. A. Puebla C. Branes M. C. Escamilla R. (2019). *De novo* expression of functional connexins 43 and 45 hemichannels increases sarcolemmal permeability of skeletal myofibers during endotoxemia. Biochim. Biophys. Acta Mol. Basis Dis. 1865 (10), 2765–2773. 10.1016/j.bbadis.2019.06.014 31228617

[B9] Cea L. A. Cisterna B. A. Puebla C. Frank M. Figueroa X. F. Cardozo C. (2013). *De novo* expression of connexin hemichannels in denervated fast skeletal muscles leads to atrophy. Proc. Natl. Acad. Sci. U. S. A. 110 (40), 16229–16234. 10.1073/pnas.1312331110 24043768 PMC3791696

[B10] Chen X. Yuan S. Mi L. Long Y. He H. (2023). Pannexin1: insight into inflammatory conditions and its potential involvement in multiple organ dysfunction syndrome. Front. Immunol. 14, 1217366. 10.3389/fimmu.2023.1217366 37711629 PMC10498923

[B11] Cisterna B. A. Vargas A. A. Puebla C. Fernández P. Escamilla R. Lagos C. F. (2020). Active acetylcholine receptors prevent the atrophy of skeletal muscles and favor reinnervation. Nat. Commun. 11 (1), 1073. 10.1038/s41467-019-14063-8 32103010 PMC7044284

[B12] Cisterna B. A. Vargas A. A. Puebla C. Saez J. C. (2016). Connexin hemichannels explain the ionic imbalance and lead to atrophy in denervated skeletal muscles. Biochim. Biophys. Acta 1862 (11), 2168–2176. 10.1016/j.bbadis.2016.08.020 27580092

[B13] Contreras L. Drago I. Zampese E. Pozzan T. (2010). Mitochondria: the calcium connection. Biochim. Biophys. Acta 1797 (6-7), 607–618. 10.1016/j.bbabio.2010.05.005 20470749

[B14] De J. B. Sharshar T. Lefaucheur J. P. Authier F. J. Durand-Zaleski I. Boussarsar M. (2002). Paresis acquired in the intensive care unit: a prospective multicenter study. JAMA 288 (22), 2859–2867. 10.1001/jama.288.22.2859 12472328

[B15] Dougnac L. A. Mercado F. M. Cornejo R. R. Cariaga V. M. Hernández P. G. Andresen H. M. (2007). Prevalencia de sepsis grave en las Unidades de Cuidado Intensivo: Primer estudio nacional multicéntrico. Rev. médica Chile 135, 620–630. 10.4067/s0034-98872007000500010 17657331

[B16] Eikermann M. Koch G. Gerwig M. Ochterbeck C. Beiderlinden M. Koeppen S. (2006). Muscle force and fatigue in patients with sepsis and multiorgan failure. Intensive Care Med. 32 (2), 251–259. 10.1007/s00134-005-0029-x 16468072

[B17] Fernández-Lorente J. Esteban A. Salinero E. Traba A. Prieto J. Palencia E. (2010). Critical illness myopathy. Neurophysiological and muscular biopsy assessment in 33 patients. Rev. Neurol. 50 (12), 718–726. 20533250

[B18] Fleischmann-Struzek C. Mellhammar L. Rose N. Cassini A. Rudd K. E. Schlattmann P. (2020). Incidence and mortality of hospital- and ICU-treated sepsis: results from an updated and expanded systematic review and meta-analysis. Intensive Care Med. 46 (8), 1552–1562. 10.1007/s00134-020-06151-x 32572531 PMC7381468

[B19] Haizlip K. M. Harrison B. C. Leinwand L. A. (2015). Sex-based differences in skeletal muscle kinetics and fiber-type composition. Physiol. (Bethesda) 30 (1), 30–39. 10.1152/physiol.00024.2014 PMC4285578 25559153

[B20] Ham D. J. Murphy K. T. Chee A. Lynch G. S. Koopman R. (2014). Glycine administration attenuates skeletal muscle wasting in a mouse model of cancer cachexia. Clin. Nutr. 33 (3), 448–458. 10.1016/j.clnu.2013.06.013 23835111

[B21] Hermans G. Van den Berghe G. (2015). Clinical review: intensive care unit acquired weakness. Crit. Care 19 (1), 274. 10.1186/s13054-015-0993-7 26242743 PMC4526175

[B22] Khan J. Harrison T. B. Rich M. M. (2008). Mechanisms of neuromuscular dysfunction in critical illness. Crit. Care Clin. 24 (1), 165–177. 10.1016/j.ccc.2007.10.004 18241784 PMC2268032

[B23] Kuznetsov A. V. Veksler V. Gellerich F. N. Saks V. Margreiter R. Kunz W. S. (2008). Analysis of mitochondrial function *in situ* in permeabilized muscle fibers, tissues and cells. Nat. Protoc. 3 (6), 965–976. 10.1038/nprot.2008.61 18536644

[B24] Laitano O. Robinson G. P. Garcia C. K. Mattingly A. J. Sheikh L. H. Murray K. O. (2021). Skeletal muscle interleukin-6 contributes to the innate immune response in septic mice. Shock 55 (5), 676–685. 10.1097/SHK.0000000000001641 32826815 PMC8607997

[B25] Lang C. H. Frost R. A. Vary T. C. (2007). Regulation of muscle protein synthesis during sepsis and inflammation. Am. J. Physiol. Endocrinol. Metab. 293 (2), E453–E459. 10.1152/ajpendo.00204.2007 17505052

[B26] Loureiro A. V. Moura-Neto L. I. Martins C. S. Silva P. I. M. Lopes M. B. S. Leitão R. F. C. (2022). Role of Pannexin-1-P2X7R signaling on cell death and pro-inflammatory mediator expression induced by Clostridioides difficile toxins in enteric glia. Front. Immunol. 13, 956340. 10.3389/fimmu.2022.956340 36072579 PMC9442043

[B27] McNair B. D. Marcello N. A. Smith D. T. Schmitt E. E. Bruns D. R. (2020). Changes in muscle mass and composition by exercise and hypoxia as assessed by DEXA in mice. Med. Kaunas. 56 (9), 446. 10.3390/medicina56090446 PMC7558449 32899136

[B28] Prauchner C. A. (2017). Oxidative stress in sepsis: pathophysiological implications justifying antioxidant co-therapy. Burns 43 (3), 471–485. 10.1016/j.burns.2016.09.023 28034666

[B29] Prest J. Nguyen T. Rajah T. Prest A. B. Sathananthan M. Jeganathan N. (2022). Sepsis-related mortality rates and trends based on site of infection. Crit. Care Explor 4 (10), e0775. 10.1097/CCE.0000000000000775 36248320 PMC9556121

[B30] Riquelme M. A. Cea L. A. Vega J. L. Boric M. P. Monyer H. Bennett M. V. (2013). The ATP required for potentiation of skeletal muscle contraction is released via pannexin hemichannels. Neuropharmacology 75, 594–603. 10.1016/j.neuropharm.2013.03.022 23583931

[B31] Rocheteau P. Chatre L. Briand D. Mebarki M. Jouvion G. Bardon J. (2015). Sepsis induces long-term metabolic and mitochondrial muscle stem cell dysfunction amenable by mesenchymal stem cell therapy. Nat. Commun. 6, 10145. 10.1038/ncomms10145 26666572 PMC4682118

[B32] Ruiz-Meana M. Abellán A. Miró-Casas E. Agulló E. Garcia-Dorado D. (2009). Role of sarcoplasmic reticulum in mitochondrial permeability transition and cardiomyocyte death during reperfusion. Am. J. Physiol. Heart Circ. Physiol. 297 (4), H1281–H1289. 10.1152/ajpheart.00435.2009 19684187

[B33] Shoji K. F. Saez P. J. Harcha P. A. Aguila H. L. Saez J. C. (2014). Pannexin1 channels act downstream of P2X 7 receptors in ATP-induced murine T-cell death. Channels (Austin) 8 (2), 142–156. 10.4161/chan.28122 24590064 PMC4048303

[B34] Singer M. Deutschman C. S. Seymour C. W. Shankar-Hari M. Annane D. Bauer M. (2016). The third international consensus definitions for sepsis and septic shock (Sepsis-3). JAMA 315 (8), 801–810. 10.1001/jama.2016.0287 26903338 PMC4968574

[B35] Tiao G. Fagan J. M. Samuels N. James J. H. Hudson K. Lieberman M. (1994). Sepsis stimulates nonlysosomal, energy-dependent proteolysis and increases ubiquitin mRNA levels in rat skeletal muscle. J. Clin. Invest 94 (6), 2255–2264. 10.1172/JCI117588 7989581 PMC330052

[B36] Trojaborg W. Weimer L. H. Hays A. P. (2001). Electrophysiologic studies in critical illness associated weakness: myopathy or neuropathy--a reappraisal. Clin. Neurophysiol. 112 (9), 1586–1593. 10.1016/s1388-2457(01)00572-7 11514240

[B37] Wasyluk W. Zwolak A. (2021). Metabolic alterations in sepsis. J. Clin. Med. 10 (11), 2412. 10.3390/jcm10112412 34072402 PMC8197843

