# Supplementary figures and images for: Connexin hemichannels and early atrophic signaling in muscle during sepsis
Source: Front Physiol. 2025 Feb 24;16:1514769. doi: 10.3389/fphys.2025.1514769 (PMC11891358; doi:10.3389/fphys.2025.1514769)

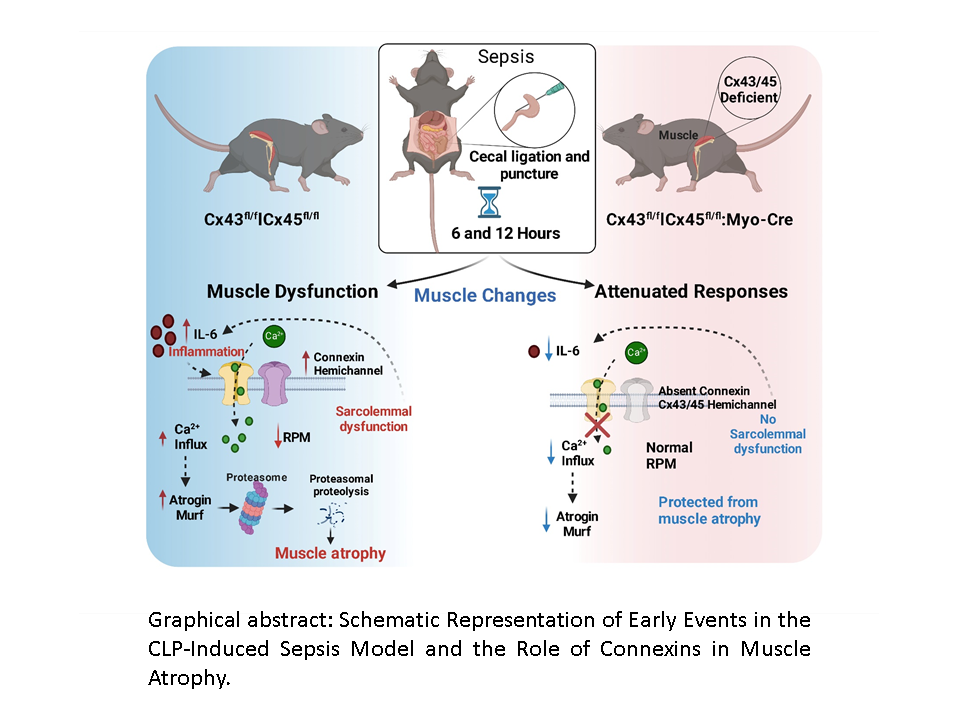

Supplement: Supplementary file 1 [file Image1.tif]
