# Supplementary material for: Connexin hemichannels and early atrophic signaling in muscle during sepsis
Source: Front Physiol. 2025 Feb 24;16:1514769. doi: 10.3389/fphys.2025.1514769 (PMC11891358; doi:10.3389/fphys.2025.1514769)
Supplement: Supplementary file 2 [file DataSheet1.pdf]

Supplementary Material

Figure 1

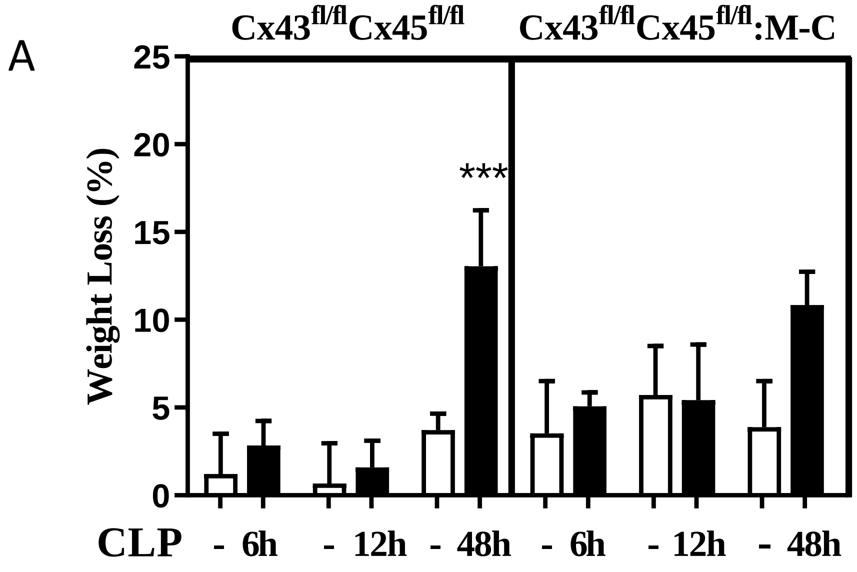

Figure 1. Animal weight changes after CLP-induced sepsis. The body weight over time in the control and at 6 and 12 and 48 hours after CLP; a sepsis model. The body weight loss in Cx43fl/flCx45fl/fl and Cx43fl/flCx45fl/fl:M-C (Cx43/45-deficient) mice. n= 4-7 (at least 4 for each group). Statistical comparisons were conducted by utilizing GraphPad Prism software and non-parametric tests, specifically the Kruskal–Wallis test followed by Dunn's multiple comparison test. The error bars represent the standard error of the mean (SEM). ***p<0,001

Figure 2

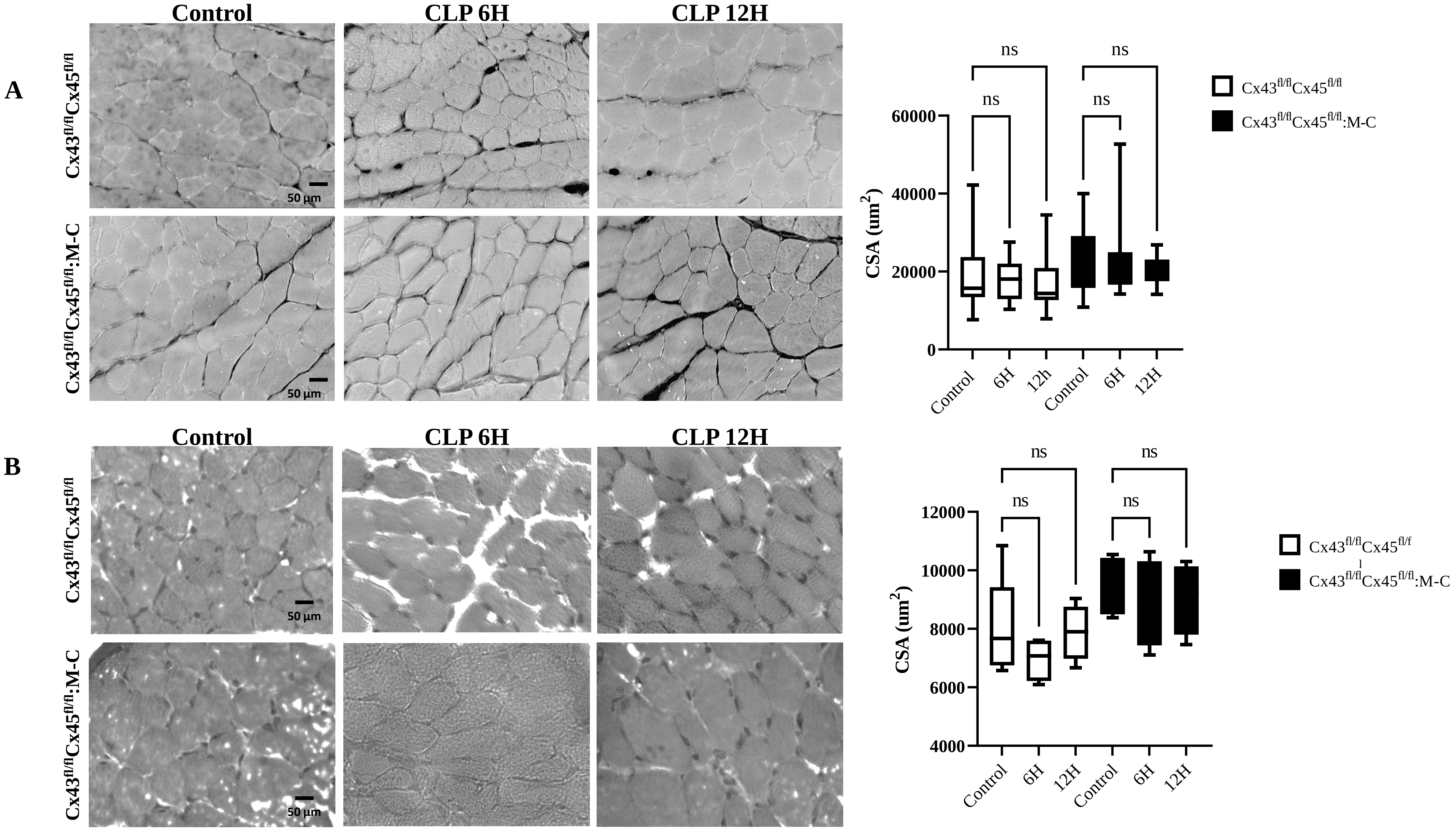
A
Control
CLP 6H
CLP 12H
Cx43fl/flCx45fl/fl
Cx43fl/flCx45fl/fl:M-C
50 µm
50 µm
ns
ns
ns
ns
60000
40000
20000
0
CSA (um2)
Control
6H
12h
Control
6H
12H
Cx43fl/flCx45fl/fl
Cx43fl/flCx45fl/fl:M-C
B
Control
CLP 6H
CLP 12H
Cx43fl/flCx45fl/fl
Cx43fl/flCx45fl/fl:M-C
50 µm
50 µm
ns
ns
ns
ns
12000
10000
8000
6000
4000
CSA (um2)
Control
6H
12H
Control
6H
12H
Cx43fl/flCx45fl/f
l
Cx43fl/flCx45fl/fl:M-C

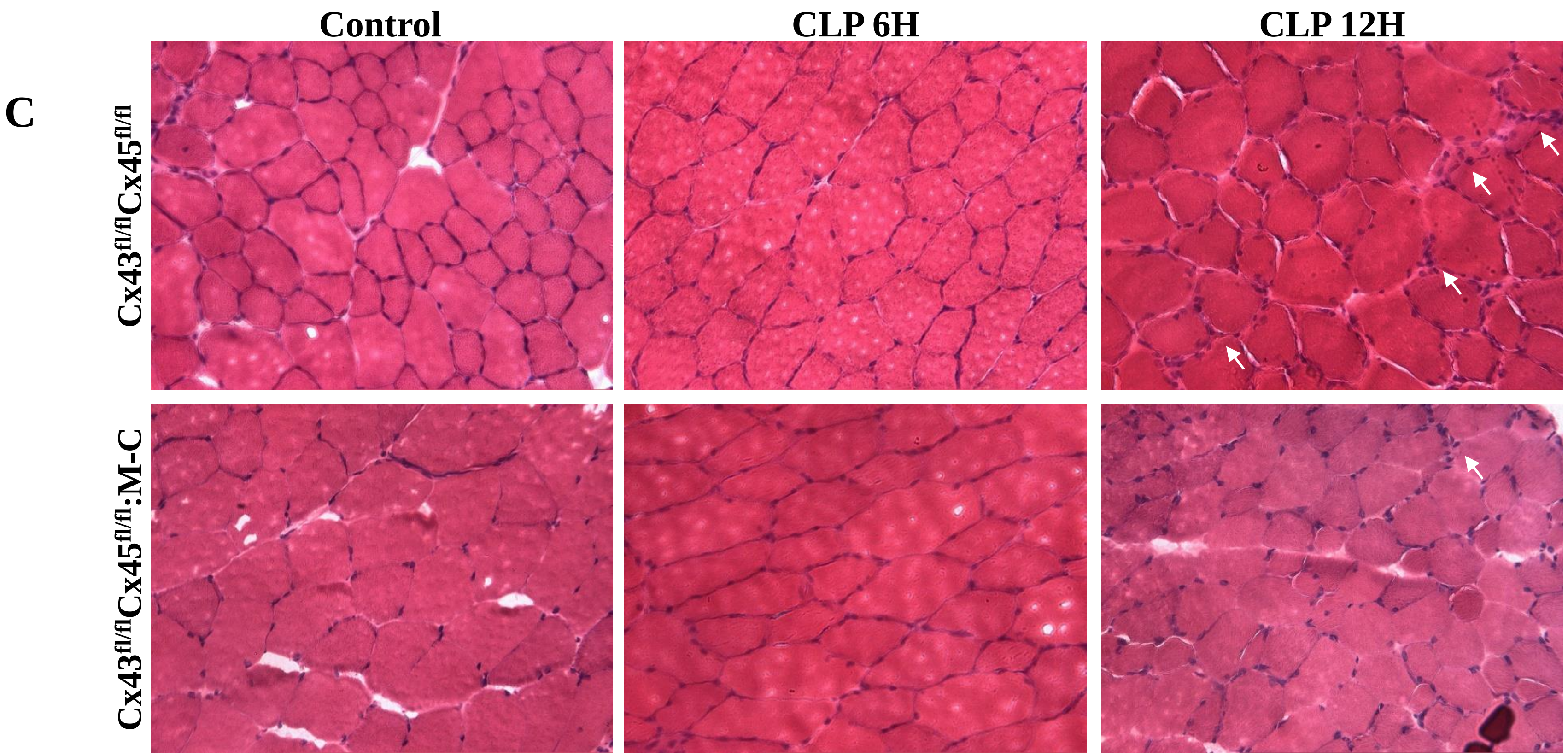

Figure 2. The CSA over time in controls, and at 6 and 12 hours after CLP, as a sepsis model. The cross-sectional area (CSA) of muscle fibers was evaluated in histological sections from mouse muscle tissue. For each mouse, at least three histological sections were analyzed, and from each section, a minimum of three random images were selected. The CSA of the fibers was quantified in each image, and the media of each fiber area was plotted (A) The tibialis anterior and (B) the diaphragm CSA in control $Cx43^{fl/fl}Cx45^{fl/fl}$ and $Cx43^{fl/fl}Cx45^{fl/fl}$:M-C (Cx43/45-deficient) mice. n=3-4 (animals) for each group . (B). The result is expressed as the min to max in a box &whiskers graph. (C) Hematoxylin and eosin staining of tibialis anterior (TA) muscle from $Cx43^{fl/fl}Cx45^{fl/fl}$ and $Cx43^{fl/fl}Cx45^{fl/fl}$:M-C (Cx43/45-deficient) mice at 0, 6, and 12 hours post-CLP, The white arrows indicate the infiltration of immune cells, suggesting an inflammatory response. Statistical comparisons were conducted by utilizing GraphPad Prism software and non-parametric tests, specifically the Kruskal–Wallis test followed by Dunn's multiple comparison test (* $p < 0.05$, ** $p < 0.01$).

Figure 3

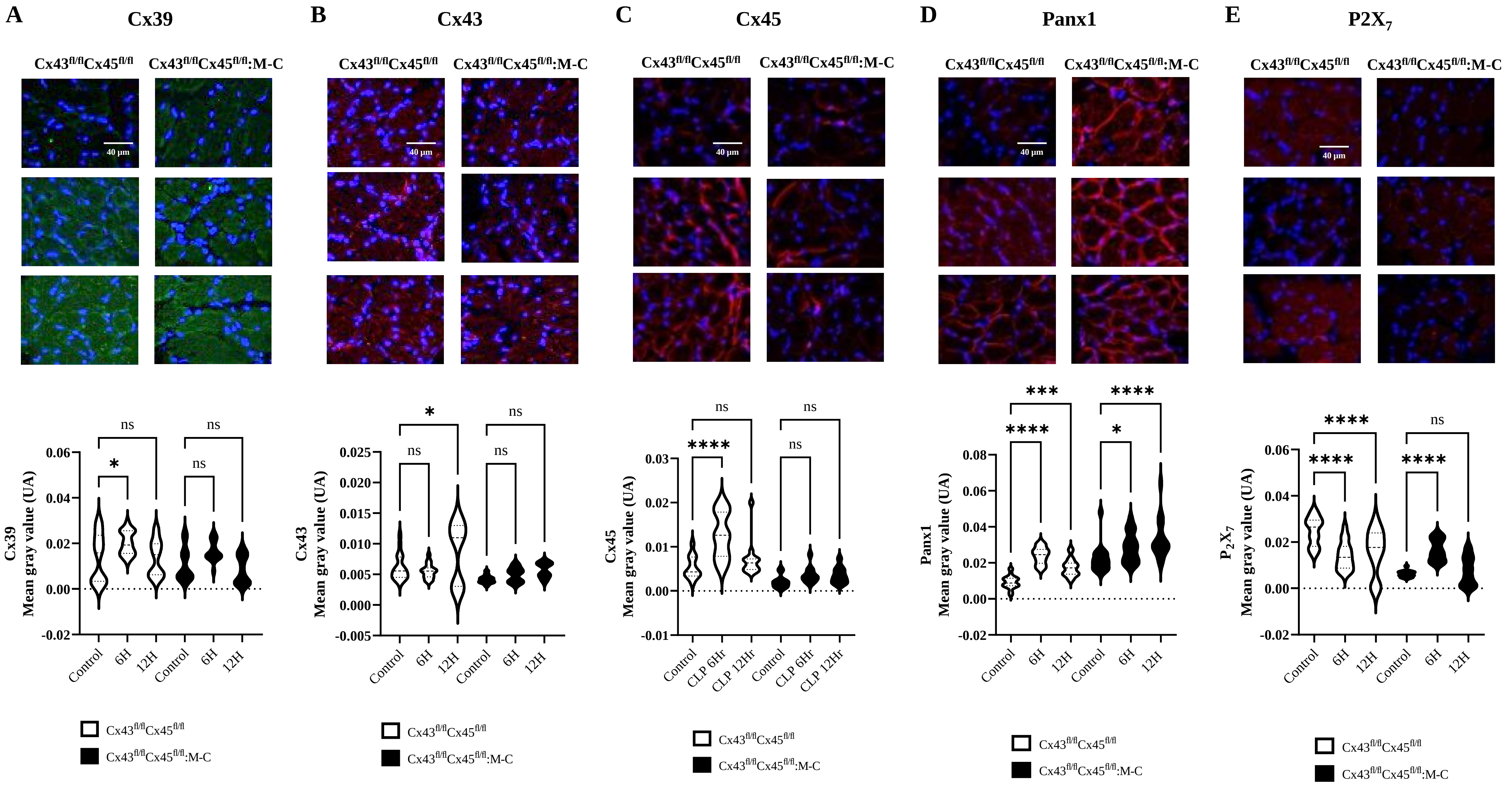

A
Cx39
B
Cx43
C
Cx45
D
Panx1
E
P2X7
Cx43fl/flCx45fl/fl
Cx43fl/flCx45fl/fl:M-C
Control
6 h
12 h
40 µm
ns
*
****
***
Cx39 Mean gray value (UA)
Cx43 Mean gray value (UA)
Cx45 Mean gray value (UA)
Panx1 Mean gray value (UA)
P2X7 Mean gray value (UA)
Control
6H
12H
CLP 6Hr
CLP 12Hr

Figure 3. Cx43/45-deficient mice are protected against the sepsis-induced de novo expression of connexin hemichannels in the sarcolemma of diaphragm muscle. Representative images and quantification of immunofluorescence for (A) Cx39, (B) Cx43, (C) Cx45, (D) Panx1, and (E) P2X7Rs in tibialis anterior muscle sections from control and CLP mice at different time points post-CLP, for Cre- ($Cx43^{fl/fl}Cx45^{fl/fl}$) and Cx43/Cx45-expression-deficient ($Cx43^{fl/fl}$ $Cx45^{fl/fl}$) mice. Fluorescence intensity was quantified in at least 3 histological sections analyzed from a minimum of 3 mice per treatment group. The data is presented using box and violin plots. Statistical comparisons were performed using GraphPad Prism software with non-parametric tests (Kruskal–Wallis test followed by Dunn's multiple comparison test). Error bars represent the standard error of the mean (SEM) (* $p < 0.05$, ** $p < 0.01$, *** $p < 0.001$).

Figure 4

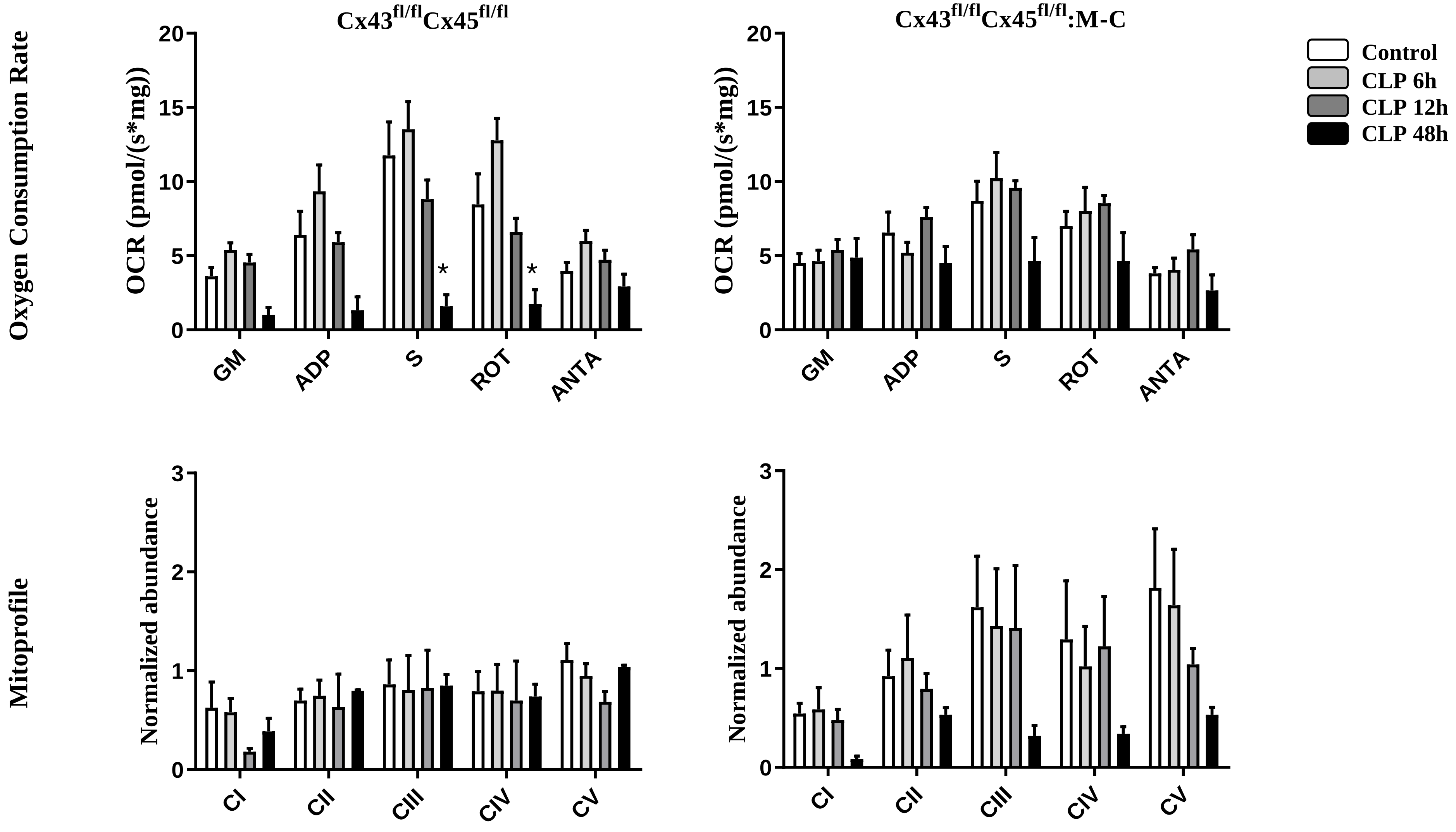

Oxygen Consumption Rate
Mitoprofile
$Cx43^{fl/fl}Cx45^{fl/fl}$
$Cx43^{fl/fl}Cx45^{fl/fl}$:M-C
OCR (pmol/(s*mg))
Normalized abundance
GM
ADP
S
ROT
ANTA
CI
CII
CIII
CIV
CV
Control
CLP 6h
CLP 12h
CLP 48h

Figure 4: The oxygen consumption rate (OCR) in permeabilized muscle fibers does not change in early sepsis. All the experiments were carried out at 6 and 12 hours after CLP in permeabilized muscle fibers from Cre- ($Cx43^{fl/fl}$ $Cx45^{fl/fl}$) and Cx43/Cx45-expression-deficient ($Cx43^{fl/fl}Cx45^{fl/fl}$:M-C) mice. Respiration was stimulated by adding the following substrates: glutamate and malate (GM, state 2, complex I), saturating ADP (state 3, complex I), succinate (S, state 3, complex I+II), rotenone (ROT, state 3, complex III), and antimycin A (ANTA, background). The average rates of oxygen consumption for each condition are shown. (A) The OCR for Cre- mice; (B) the OCR for Cx43/45 deficient mice; (C) the normalized abundance of mitochondrial complexes for Cre- mice; and (D) the normalized abundance of mitochondrial complexes for Cx43/45-deficient mice. A statistical evaluation of the differences between the control and CLP for each group was performed using a one-/two-way ANOVA test. The results are expressed as the mean $\pm$ SEM.
